# Sanatorium Treatment of Tuberculosis

**Published:** 1921-01-22

**Authors:** 


THE PUBLIC WELL-BEING—(*continued*).

### Sanatorium Treatment of Tuberculosis.

### THE CAUSE OF DISAPPOINTING RESULTS.

Some weeks ago attention was called in these columns to the importance of early diagnosis in cases of tuberculosis, and the difficulty of securing good results without a much more effective co-ordination of the methods employed by public-health committees, and an increase in the number of efficient tuberculosis officers. This view is certainly emphasised by the nature of the reports which have been published during the past year on the results of sanatorium treatment. To take one report, quoted and commented upon in a recent issue of the *Morning Post*, the County of London Insurance Committee state that in 1919 there were 1,484 ex-Service men, 690 civilian males, and 761 females discharged from sanatoria after a course of treatment provided by the Committee.

Of these patients a considerable number were not restored to health at the time of discharge. In the first group 699, in the second 294, and in the third 293 patients were reported to be unfit for work on leaving the sanatorium. That is to say, out of 2,905 people who were sent to sanatoria 1,286, or 43 per cent., were unfit to work on discharge. Moreover, in that year seventy-five patients actually died in sanatoria. If the figures are taken over a series of years, the results are even less satisfactory. Of all patients whose treatment was commenced by the London Insurance Committee in 1914 over 70 per cent. died before the end of 1918. These figures, it is scarcely necessary to point out, are not encouraging to health officials or to the general public, for sanatoria are admittedly an expensive form of treatment, and it will be a serious matter if the taxpayers and ratepayers are driven to the conclusion that they are a failure. Under the best practical conditions we know that they are a very important factor in the fight against tuberculosis. For nearly half a century evidence has been accumulated to show beyond doubt that pulmonary tuberculosis may be arrested by sanatorium treatment in over 90 per cent. of cases, "provided, firstly, that the disease is detected and treated in its early stages, and, secondly, that the treatment is sufficiently prolonged."

These are two essential considerations which go to the root of the matter. How far are the conditions observed under the "sanatorium benefit" of the Insurance Act? The truth seems to be that the unsuitable cases are apt to be sent to sanatoria, because a large number of patients do not seek advice until the disease is well advanced, and many doctors have not the special qualifications necessary for making a firm diagnosis at the beginning of the disease. Tuberculosis is a notifiable disease, and every doctor, when he has diagnosed a case, must report the fact to the local medical officer of health. When a patient dies his death and the cause thereof are notified to the Registrar. "It is therefore possible to determine what time has elapsed between the discovery of the disease and the death of the patient. Tuberculosis is known to be a disease of many years duration, but the interval between the notification and death of individual patients is too often only a few months, or even weeks."

The writer in the *Morning Post* comes, therefore, to the inevitable conclusion that early diagnosis is the key to the situation. We agree with the contention also that the average duration of sanatorium treatment—about twelve weeks—allowed by insurance committees throughout the country is too short, and we may add that the waiting period is too long. Nearly everywhere the period is from seven to fifteen days for an ex-soldier, twenty-eight days on the average for a civilian adult, and from eight to twelve weeks for a child. A critical examination of the London boroughs has shown, as we have pointed out on a former occasion, that they have from 37 to 455 deaths from tuberculosis, and that, on the other hand, a single physician can do useful work only if he has charge of a population with not more than 120 such deaths in a year. For the sake of economy and convenience Dr. F. N. K. Menzies, in an official analysis of the position, accepted a maximum figure of 160. On that basis London would require not thirty-two but fifty whole-time tuberculosis officers—that is to say, an average of one per 90,000 inhabitants. Their work would be enormous even then, as they would each have to supervise some 500 to 600 consumptives and their contacts, or possibly 1,000 patients. But the result would be correlated effort and greater efficiency, because the chances of detecting disease early and of preventing its communication to others would be immensely increased. The total expenditure in respect of the London boroughs would, it is estimated, be no more than £391,000.

